# Prognostic Performance of C-Reactive Protein for Tuberculosis Outcome: Protocol for a Systematic Review and Meta-Analysis

**DOI:** 10.2196/80744

**Published:** 2026-06-16

**Authors:** Jessica Edelyne, Muhammad Prasetio Wardoyo, Luthfiyah Zanida Putri, Salsabila Hulwani, Assica Permata Amalya Hakiman, Yogik Onky Silvana Wijaya, Erlina Burhan

**Affiliations:** 1 Master in Biomedical Sciences, Faculty of Medicine, Public Health, and Nursing, Universitas Gadjah Mada, Senolowo, Jl. Farmako, Sekip Utara, Kec Depok, Kabupaten Sleman, Daerah Istimewa Yogyakarta, 55281, Indonesia, 62 274-560-300; 2 Faculty of Pharmacy, Université de Montpellier, Montpellier, France; 3 Respiratory Programmatic Implementation and Research Institute, Jakarta Timur, Indonesia; 4 Clinical Trials Unit, University College London, London, United Kingdom; 5 Department of Biochemistry, Faculty of Medicine, Public Health, and Nursing, Universitas Gadjah Mada, Yogyakarta, Indonesia; 6 Department of Pulmonology and Respiratory Medicine, Faculty of Medicine, University of Indonesia - Persahabatan Hospital, Jakarta Timur, Indonesia

**Keywords:** tuberculosis, C-reactive protein, CRP, prognostic, mortality, death, systematic review, meta-analysis, Preferred Reporting Items for Systematic Reviews and Meta-Analyses Protocols, PRISMA

## Abstract

**Background:**

Tuberculosis (TB) remains a major global health challenge, with substantial mortality despite the availability of standardized treatment regimens. Accurate prognostication remains difficult, as no host-derived biomarker is routinely used to predict TB outcomes. C-reactive protein (CRP), a widely available acute-phase reactant, has been proposed as a potential prognostic biomarker, but its prognostic value for mortality in TB has not been systematically synthesized.

**Objective:**

This systematic review and meta-analysis aimed to evaluate the prognostic value of baseline CRP in predicting mortality among adult patients with TB and to examine whether the reported association varies across relevant clinical subgroups and study characteristics.

**Methods:**

This protocol was developed in accordance with PRISMA-P (Preferred Reporting Items for Systematic Review and Meta-Analysis Protocols) guidelines. Eligible studies included full-text English-language cohort studies, observational studies, case-control studies, and control arms of randomized controlled trials involving adults (aged 18 years and older) with microbiologically confirmed pulmonary or extrapulmonary TB. Included studies were required to assess baseline CRP at or near the time of TB diagnosis and to report mortality using quantitative effect estimates such as hazard ratios, odds ratios, or risk ratios. Studies limited to pediatric or latent TB populations and nonoriginal publication types were excluded. The literature search was conducted in the Cochrane Library, PubMed, Scopus, MEDLINE via Ovid, ProQuest, and medRxiv. Risk of bias was assessed using the QUIPS (Quality in Prognosis Studies) tool, and certainty of evidence was assessed using GRADE (Grading of Recommendations Assessment, Development, and Evaluation). A structured qualitative synthesis was undertaken for all included studies, and a random-effects meta-analysis was performed where studies were sufficiently comparable.

**Results:**

The protocol was prospectively registered in PROSPERO (CRD420251101984) in July 2025. The electronic search covered the period from database inception to June 30, 2025, and was conducted from July 10, 2025, to July 14, 2025. Screening, full-text assessment, data extraction, methodological appraisal, and evidence synthesis were completed. A full manuscript reporting the findings of the completed systematic review and meta-analysis was subsequently prepared.

**Conclusions:**

This protocol provides a transparent record of the rationale, objectives, and methodological approach used to evaluate CRP as a prognostic biomarker for TB mortality. The completed review is expected to inform the interpretation of CRP in TB care, identify evidence gaps, and guide future prognostic research.

## Introduction

### Rationale

Tuberculosis (TB) still remains a public health concern today due to its significant morbidity and mortality. Globally, as many as 10.8 million people annually fall ill with TB and 1.25 million people die because of it, as reported in the Global Tuberculosis Report 2024 released by the World Health Organization (WHO) [1]. Although progress has been made with the introduction of standardized regimens for TB treatment, especially shorter regimens for drug-susceptible TB and drug-resistant TB [2], the high annual mortality rate caused by TB still triggers discussions on how to improve the management of patients with TB for better outcomes. One of the discussions currently underway concerns the prognostication of TB outcomes.

Predicting TB outcomes remains complex and challenging, influenced by diverse host and environmental factors [3,4]. While TB treatment has largely focused on bacteriological indicators, the role of the host immune response in determining clinical outcomes remains underexplored [5,6]. Currently, no well-established prognostic marker is routinely used to reflect host status and predict outcomes in patients with TB, limiting clinicians’ ability to implement individualized care plans [7,8]. Identifying accessible and reliable prognostic indicators is, therefore, essential to support early intervention and improve survival.

Among various host-derived biomarkers, C-reactive protein (CRP) has emerged as a promising candidate. CRP is a well-known acute-phase reactant that increases in response to inflammation, including TB infection. It is widely accessible, low-cost, and easy to measure, even in resource-limited settings [9,10]. CRP levels are frequently elevated in active TB and decline with successful treatment [11]. This is found to be true even in the case of extrapulmonary TB, such as spinal TB, where CRP levels decreased significantly postoperatively, supporting its use in evaluating rehabilitation [12].

Beyond treatment monitoring, CRP has been identified as an independent prognostic factor for severe disease forms. In patients with pulmonary TB and respiratory failure, nonsurvivors had significantly higher CRP levels than survivors [13]. High CRP levels were associated with other poor prognostic markers, including elevated APACHE II scores, low PaO_2_, and hypoalbuminemia, highlighting CRP’s potential as an early warning marker for clinical deterioration [13].

Nevertheless, CRP is not specific to TB and may be elevated in other infectious or inflammatory conditions [14]. One study found no significant difference in CRP levels between patients with and without sputum conversion after 2 months of treatment [15], and its overlap with conditions like community-acquired pneumonia further complicates its diagnostic value. However, when combined with other clinical indicators, CRP may still contribute valuable prognostic insight.

The WHO TB Knowledge Sharing Platform currently recognizes CRP as a valuable triage and prognostic biomarker. A CRP threshold of ≥5 mg/L is recommended to support TB screening and risk stratification in people with HIV, while a higher cut-off of ≥10 mg/L may offer better specificity in individuals who are HIV-negative [9]. CRP levels may differ by TB disease site, suggesting the need to evaluate performance across clinical subgroups [16,17]. These findings suggest the need to evaluate the prognostic performance of CRP across clinical subgroups, including TB with versus without HIV and pulmonary versus extrapulmonary TB.

Given the biological plausibility, accessibility, and growing evidence of CRP’s prognostic value in TB, a systematic and comprehensive synthesis is warranted. This systematic review and meta-analysis, titled “Prognostic Performance of C-Reactive Protein for Tuberculosis Outcome (PROSPECT-TB SR-MA),” aims to clarify the utility of CRP in predicting mortality among adult patients with TB. Subgroup analyses will be conducted based on HIV coinfection status and TB type (pulmonary vs extrapulmonary).

### Aim and Objectives

This systematic review aims to evaluate the prognostic value of CRP in predicting mortality among adult patients with TB. Specifically, this systematic review will systematically identify, summarize, and appraise existing studies that assess CRP as a prognostic biomarker in TB. It will also examine the methodologies used to measure CRP, analyze the strength and consistency of its association with mortality outcomes, and assess the clinical applicability of CRP-based prognostication across various populations and health care settings.

### Research Questions

The main research question for this systematic review and meta-analysis was formulated using the domain, determinants, and outcome approach (Table 1): among adult patients with TB, does baseline CRP have prognostic value in predicting mortality?

**Table 1.** Review question framework based on the domain, determinants, and outcome approach.

Aspects	Component of interest	Criteria
Domain	Adult patients with TB^a^	The population of interest comprised adult patients (18 years and older) diagnosed with TB, regardless of manifestation (pulmonary or extrapulmonary) and comorbidities (eg, HIV, DM^b^).
Determinants	CRP^c^	This review included studies that assess CRP as a prognostic factor, with CRP measured when the patients are diagnosed through any standard laboratory method. Studies ought to report a quantitative association between CRP levels and mortality.
Outcome	Mortality	The outcome of interest is mortality, including all causes of mortality as defined by the primary studies. While all-cause mortality was extracted to ensure inclusiveness, subgroup or sensitivity analyses might be conducted (if data allows) to assess the prognostic value of CRP in TB-related versus non–TB-specific causes of death.

a TB: tuberculosis.

b DM: diabetes mellitus.

c CRP: C-reactive protein.

Along with this main question, 2 secondary questions were asked in this review:

1. What are the direction and magnitude of the reported association between baseline CRP and mortality across the included studies?
2. Where data permit, does the reported association vary according to clinical subgroups or study characteristics, such as HIV status, TB type, CRP threshold, or other methodological features?

## Methods

### Study Design

This protocol was developed in accordance with the PRISMA-P (Preferred Reporting Items for Systematic Review and Meta-Analysis Protocols) guidelines (Checklist 1) [18]. The systematic review and meta-analysis were designed in accordance with the methodological guidance for prognostic factor reviews as outlined by Riley et al [19]. The final review report will follow the PRISMA 2020 statement to ensure transparent and comprehensive reporting.

### Eligibility Criteria

This systematic review included studies involving adult patients aged 18 years or older with microbiologically confirmed TB, including pulmonary or extrapulmonary disease. Eligible studies were required to assess baseline CRP measured at or near the time of TB diagnosis and to report mortality outcomes using quantitative effect estimates, such as hazard ratios (HRs), odds ratios (ORs), or risk ratios (RRs).

Eligible study designs included prospective or retrospective cohort studies, observational studies, case-control studies, and control arms of randomized controlled trials. Only full-text articles published in English were considered.

Studies were excluded if they did not report mortality as an outcome, assessed CRP without evaluating its prognostic association with mortality, were conducted exclusively in pediatric populations, focused on latent TB, or were case reports, case series, conference abstracts, letters, correspondences, narrative reviews, expert commentaries, protocols, editorials, or in vitro or animal studies. For duplicate or overlapping publications, the most complete or methodologically relevant report was prioritized.

### Information Sources

The literature search was conducted across the following electronic databases: Cochrane Library, PubMed, Scopus, MEDLINE via Ovid, ProQuest, and medRxiv. No date restrictions were applied. Only studies published in English were considered. Forward and backward citation tracking of included studies and relevant review articles was also performed to identify additional eligible records.

### Search Strategy

The search strategy combined free-text keywords and controlled vocabulary terms related to TB, CRP, prognosis, and mortality. Search terms included, but were not limited to, “tuberculosis,” “TB,” “C-reactive protein,” “CRP,” “mortality,” “death,” and “prognosis,” combined using Boolean operators (AND/OR). The search covered the period from database inception to June 30, 2025, and the electronic search was conducted from July 10, 2025, to July 14, 2025. Detailed search strategies are provided in Multimedia Appendix 1.

The search process was carried out by members of the review team, and any uncertainties regarding eligibility were resolved during the screening and full-text assessment stages through discussion and reviewer consensus. No additional search updates were undertaken after the final search date prior to the completion of the review.

### Study Records

#### Data Management

All identified references were managed using Rayyan (rayyan.ai). Duplicate records were identified and removed before the title and abstract screening. Review records were stored in structured extraction files for subsequent appraisal and synthesis.

#### Selection Process

Three reviewers (JE, LZP, and SH), working in pairs, independently screened titles and abstracts of the retrieved studies against the prespecified eligibility criteria. Studies considered potentially eligible then underwent a full-text assessment. In cases of disagreement, additional reviewers (MPW and APAH) were consulted, and discrepancies were resolved through discussion until consensus was reached.

#### Data Collection Process

Data extraction was performed using a standardized extraction form informed by the CHARMS-PF (Critical Appraisal and Data Extraction for Systematic Reviews of Prediction Modelling Studies for Prognostic Factor) checklist [19] for prognostic factor reviews. Emphasis was placed on extracting adjusted effect estimates where available because these are most informative for evaluating the independent prognostic value of CRP after accounting for potential confounders.

The extraction process involved multiple reviewers. Study data were extracted and checked independently by members of the review team, and discrepancies were resolved through discussion and consensus. Where overlapping study populations were suspected, authors were contacted to clarify potential duplication and to request additional adjusted effect estimates where relevant. However, only limited additional information was obtained, and no extra usable data were incorporated.

Where data were missing, unclear, or incompletely reported, this was documented and considered during the risk-of-bias assessment and interpretation of the evidence. CRP values reported in different units were converted to a common unit, where necessary, to support comparability across studies.

### Data Items

Data extraction was guided by the review questions and planned synthesis. The following information was extracted from each included study: study characteristics (author, year of publication, country, study design, setting, sample size, and follow-up duration), participant characteristics (age, sex, TB type, HIV status, and relevant comorbidities where available), CRP-related variables (timing of measurement, assay method, unit, and threshold or cut-off value), outcome definitions (all-cause mortality and, where reported, cause-specific mortality), and analytic variables, including reported effect measures, covariates used for adjustment, subgroup-specific findings, and reported prognostic performance measures where available (Table 2).

**Table 2.** Information for data extraction, subsequent summary, and appraisal.

Data	Key items
Source of data	Study design (eg, prospective and retrospective cohort, observational study, control arm of RCT^a^) Whether the study was registered (eg, PROSPERO, ClinicalTrials.gov)
Participants	Eligibility criteria and recruitment methods (number of studies, inclusion and exclusion criteria) Age and sex of participants Type of TB^b^ (pulmonary or extrapulmonary) HIV coinfection status Drug sensitivity status (DR-TB^c^ and DS-TB^d^) Duration of follow-up: time to event (mortality)
CRP^e^ measurement (determinant)	Timing of CRP measurement (during diagnosis) Measurement method (eg, quantitative assay, unit used) CRP cutoff or threshold values (eg, ≥5 mg/L, ≥10 mg/L)
Outcomes to be predicted	All-cause mortality Whether outcome assessors were blinded to CRP status
Subgroup characteristics	Availability of data for subgroups: TB with versus without HIV Pulmonary versus extrapulmonary TB MDR-TB^f^ versus XDR-TB^g^ CRP as continuous versus dichotomous variable
Sample size	Total number of participants Whether sample size calculation was reported
Missing data	Whether missing data were reported for CRP or outcome Method of handling missing data (eg, complete case analysis, imputation, or other methods)
Model performance	Sensitivity, specificity, or predictive values CRP cutoff performance (eg, ≥5 mg/L or ≥10 mg/L) Calibration metrics (eg, observed vs expected)
Results	Main effect estimates (OR^h^, HR^i^, RR^j^ with 95% CI) CRP levels significantly associated with mortality Subgroup findings (eg, HIV status, TB type) Key statistical conclusions from each study
Interpretation and discussion	Study authors’ interpretation of findings Clinical implications and potential utility of CRP Study authors’ reported strengths and limitations Recommendations for practice or future research

a RCT: randomized controlled trial.

b TB: tuberculosis.

c DR-TB: drug-resistant tuberculosis.

d DS-TB: drug-susceptible tuberculosis.

e CRP: C-reactive protein.

f MDR-TB: multidrug-resistant tuberculosis

g XDR-TB: extended drug–resistant tuberculosis

h OR: odds ratio.

i HR: hazard ratio.

j RR: risk ratio.

These extracted data were used to support both qualitative and quantitative synthesis. Study and participant characteristics were used to describe the included evidence base and to explore clinical and methodological heterogeneity. Effect estimates and corresponding CIs were used for meta-analysis where studies were sufficiently comparable. Variables such as HIV status, TB type, CRP threshold, timing of CRP measurement, and follow-up duration were used to inform subgroup or sensitivity analyses where data permitted.

### Follow-Up Duration Consideration

Duration of follow-up was extracted for each included study. This is a critical contextual variable, as short follow-up periods may fail to capture mortality events and underestimate the prognostic value of CRP. If available, the time to mortality or the timing of outcome ascertainment was recorded to support time-to-event interpretation and sensitivity analyses.

### Outcomes and Prioritization

The main outcome assessed in this study is mortality.

### Risk of Bias in Individual Studies

Risk of bias was assessed independently by 2 reviewers using the QUIPS (Quality in Prognosis Studies) tool, which was used to assess the risk of bias across several key domains: study participation and attrition, measurement of prognostic factors, measurement of outcomes, adjustment for other prognostic factors, and statistical analysis and reporting. To ensure consistency in judgments, predefined criteria for evaluating signaling items within each domain were applied.

The certainty of evidence was assessed using the GRADE (Grading of Recommendations Assessment, Development, and Evaluation) tool. Each outcome began with a starting level of certainty, and then downgrading or upgrading criteria were applied according to GRADE guidance to determine the final certainty rating. Disagreements between reviewers were resolved through discussion or, if necessary, through consultation with a third reviewer.

We also specifically assessed the risk of bias due to missing data and the handling of CRP measurements across studies. If applicable, we documented whether the authors accounted for missing predictor or outcome data using imputation methods or sensitivity analyses.

The results of the risk-of-bias assessments were summarized in both tabular and narrative forms and were used to guide subgroup analyses and sensitivity analyses during the data synthesis phase. The influence of high-risk studies was examined by excluding them in secondary analyses.

### Assessing the Prognostic Performance of CRP

The prognostic value of baseline CRP for mortality in TB was assessed primarily through reported measures of association between CRP and mortality outcomes, including HRs, ORs, and RRs. Where available, additional prognostic performance measures, such as discrimination or calibration, were also extracted and summarized descriptively.

Because CRP thresholds, timing of measurement, and patient populations varied across studies, the review also examined whether the reported association between CRP and mortality differed according to relevant clinical and methodological characteristics. These characteristics included HIV status, TB type (pulmonary vs extrapulmonary), CRP threshold or categorization, and follow-up duration, where reported. Where subgroup-specific data were too limited for pooled analysis, findings were summarized narratively.

### Synthesis

A structured qualitative synthesis was undertaken for all included studies. Study characteristics, participant profiles, CRP measurement approaches, mortality definitions, and key findings were summarized in tables and narrative form.

Where at least 2 studies were sufficiently comparable in terms of population, CRP definition, outcome definition, and reported effect measure, quantitative synthesis was performed using random-effects meta-analysis. Effect estimates were synthesized separately according to the type of measure reported, such as HRs, ORs, and RRs. Where available, adjusted estimates were prioritized over unadjusted estimates for evaluating the independent prognostic value of CRP, although both were summarized separately when appropriate.

Subgroup analyses were conducted where data permitted to explore potential sources of heterogeneity, including HIV status, TB type, CRP threshold or categorization, and other relevant study characteristics. Sensitivity analyses were undertaken where appropriate to assess the robustness of pooled estimates, including the exclusion of studies at higher risk of bias or with methodological features likely to contribute substantially to heterogeneity. If studies were too heterogeneous in design, CRP categorization, outcome definition, or reported effect measure, their findings were synthesized narratively rather than pooled quantitatively.

### Meta-Biases

If at least 10 studies were available for a given meta-analysis, funnel plots and the Egger test were used to explore possible small-study effects. These analyses were interpreted cautiously because asymmetry may reflect several causes, including publication bias, methodological differences, or true heterogeneity. If fewer than 10 studies were available, formal assessment of small-study effects was not undertaken because such methods are unlikely to provide reliable results.

### Confidence in Cumulative Evidence

The overall certainty of the evidence was assessed using the GRADE approach.

### Ethical Considerations

Ethics approval was not required for this systematic review because it involved analysis of data from previously published studies and did not involve primary data collection or direct participation of human subjects. The protocol was prospectively registered in PROSPERO (CRD420251101984).

## Results

The protocol for this systematic review and meta-analysis was registered in PROSPERO (CRD420251101984) in July 2025. The electronic literature search covered the period from database inception to June 30, 2025, and was conducted from July 10, 2025, to July 14, 2025. The databases searched were the Cochrane Library, PubMed, Scopus, MEDLINE via Ovid, ProQuest, and medRxiv.

Following the completion of the search, retrieved records underwent deduplication and title or abstract screening using Rayyan. Full-text eligibility assessment was subsequently completed in accordance with the prespecified inclusion and exclusion criteria. Data extraction and methodological quality appraisal were then undertaken using the prespecified review procedures.

The evidence synthesis for the systematic review and meta-analysis was also completed. A full manuscript reporting the findings of the completed review was prepared. In the completed review, the final study selection process, characteristics of the included studies, risk-of-bias assessment, and quantitative synthesis are reported in full. This protocol manuscript, therefore, serves as a transparent record of the review rationale, objectives, and prespecified methodological approach.

## Discussion

### Expected Results

This systematic review was designed to clarify whether elevated CRP is consistently associated with higher mortality risk among adults with active TB and whether this association remains after adjustment for key clinical confounders. Particular attention was given to potential variation by HIV status, TB phenotype, timing of measurement, and CRP thresholds used across studies. These considerations were intended to inform whether CRP has sufficient standalone prognostic utility or is better positioned as one component within broader risk stratification approaches.

### Clinical Relevance of CRP in TB

CRP is widely recognized as a low-cost and accessible inflammatory biomarker [9,10], yet its role as a prognostic factor in TB management has not been definitively established. Accurate estimation of mortality risk using CRP could assist clinicians in early risk stratification, tailoring treatment monitoring and optimizing resource allocation, particularly in resource-limited and high-burden TB settings [11,20].

By identifying, summarizing, and critically appraising published studies that evaluate CRP’s prognostic performance, this systematic review served as a comprehensive resource for clinicians, policymakers, and researchers engaged in TB control efforts. Although many observational studies have explored the relationship between CRP levels and TB outcomes [11,13,20], few have followed rigorous prognostic research standards, leading to methodological biases, inadequate adjustment for confounders, and inconsistent reporting of performance metrics such as discrimination and calibration [21]. This systematic review synthesized these findings and assessed their validity and clinical applicability, using structured risk of bias and evidence quality tools.

### Comparison to Prior Work

Prior studies examined CRP in relation to TB outcomes, but the reported associations varied across populations and clinical contexts. Inconsistencies may reflect differences in baseline disease severity, HIV coinfection, timing of CRP measurement, cutoff values, outcome definitions, and the extent of confounding control. In addition, studies often differ in how they report prognostic performance (eg, reliance on association measures without clear evaluation of discrimination, calibration, or clinical utility). By applying structured risk-of-bias tools and systematically extracting prognostic factor metrics, this review synthesized existing evidence more transparently and helped to distinguish between true clinical signals and methodological or contextual sources of variability.

### Implications for Future Prognostic Research

Unlike prediction models that integrate multiple variables, this review focused on a single biomarker, allowing for a more direct comparison of its standalone prognostic value. Nevertheless, the findings may guide future research that integrates CRP into more complex prognostic models for TB.

### Dissemination Plan

We plan to disseminate the findings through peer-reviewed publications and presentations at scientific conferences. Where feasible, we will also share a concise summary of the results with TB clinicians and program stakeholders to support the interpretation of CRP in routine care and to inform future prognostic research priorities.

The findings of the completed review may help inform clinicians, policymakers, and researchers about the current evidence on the prognostic value of CRP in predicting mortality among patients with TB. They may also help contextualize the applicability of CRP across different clinical settings, particularly in resource-limited environments where CRP testing is more accessible than other biomarkers, and guide future work on risk stratification and CRP-informed clinical decision-making.

### Strengths

Strengths of this study include its adherence to the PRISMA 2020 guidelines and its alignment with relevant recommendations for conducting prognostic factor systematic reviews, such as the CHARMS framework [19,22]. Independent, duplicate review processes and a consensus-based analytical approach ensured the robustness and credibility of data interpretation. The findings of this review are expected to have practical implications for TB triage strategies, prognostic scoring systems, and individualized patient management in both high-resource and low-resource settings.

### Limitations

Some limitations of this study should be acknowledged. We anticipate substantial heterogeneity across studies in terms of CRP measurement methods, cutoff thresholds, timing of assessment, outcome definitions, and population characteristics, such as HIV status, type of TB (pulmonary or extrapulmonary), and health care settings. These variations may prevent meaningful data pooling and limit the feasibility of generating aggregate prognostic effect estimates.

Although many of the included studies may report adjusted effect estimates and have undergone internal or external validation, the clinical applicability and generalizability of their findings remain limited without standardized protocols for CRP testing and interpretation. Differences in study design, duration of follow-up, and regional health system capacities may further affect comparability. Another limitation is that, although authors of overlapping studies were contacted to clarify potential duplication and request additional adjusted effect estimates where relevant, only limited additional information was obtained. Consequently, some missing or unclear information remained and was documented as part of the risk of bias and certainty of evidence assessments.

Despite these limitations, the review will provide valuable insights into the current landscape of CRP as a prognostic biomarker in TB and will help guide future clinical research and policymaking efforts.

### Clinical Implications and Future Directions

From a clinical perspective, CRP represents an attractive candidate prognostic biomarker due to its wide availability, low cost, and routine use in TB care. However, given the variability in existing studies, this review will evaluate whether CRP has sufficient and consistent prognostic performance to be interpreted as a standalone marker for mortality risk or whether it is best used as part of a broader risk stratification approach. Integrating CRP with clinical variables such as age, HIV status, disease severity, nutritional status, and microbiological findings may improve prognostic accuracy and better inform clinical decision-making.

Future research should focus on developing and validating multimodal prognostic models incorporating CRP alongside other clinical and laboratory parameters. In addition, an individual participant data meta-analysis would be particularly valuable to harmonize CRP measurement and cutoff values, allow consistent adjustment for key confounders, and explore effect modification across important subgroups. Such approaches could support the translation of CRP-based prognostic assessment into clinically actionable tools for TB management.

### Conclusion

This protocol describes the rationale and methodological approach used to systematically review and critically appraise the prognostic value of CRP for mortality among adults with TB. By evaluating study quality, sources of heterogeneity, and (where reported) prognostic performance metrics, the review will clarify whether CRP provides clinically meaningful mortality risk information across settings and patient subgroups. The results are expected to inform the interpretation of CRP in TB care, identify evidence gaps, and guide the future development of robust prognostic models and standardized approaches to CRP measurement and reporting.

## Supplementary material

10.2196/80744 Multimedia Appendix 1 Search strategy.

10.2196/80744 Checklist 1 PRISMA-P checklist.

## Abbreviations

CHARMS: Critical Appraisal and Data Extraction for Systematic Reviews of Prediction Modelling Studies
CRP: C-reactive protein
GRADE: Grading of Recommendations Assessment, Development, and Evaluation
HR: hazard ratio
OR: odds ratio
PRISMA-P: Preferred Reporting Items for Systematic Review and Meta-Analysis Protocols
QUIPS: Quality in Prognosis Studies
RR: risk ratio
TB: tuberculosis
