# Supplementary material for: Prognostic Performance of C-Reactive Protein for Tuberculosis Outcome: Protocol for a Systematic Review and Meta-Analysis
Source: JMIR Res Protoc. 2026 Jun 16;15:e80744. doi: 10.2196/80744 (PMC13271584; doi:10.2196/80744)
Supplement: Multimedia Appendix 1 [file resprot-v15-e80744-s001.docx]

### **Appendix 2 – Search Strategy**

#### **Table S2. Search Strategy - PubMed**

#### DATE: 10/07/25; Retrieved data: 516

| Query # | Query Terms |
| --- | --- |
| 1 | (“tuberculosis” OR “TB”) |
| 2 | (“C-reactive protein” OR “C reactive protein” OR “CRP”) |
| 3 | (“prognosis” OR “mortality” OR “death” OR “survival analysis”) |
| 4 | #1 AND #2 AND #3 |

#### **Table S3. Search Strategy - Scopus**

#### DATE: 10/07/25; Retrieved data: 283

| Query # | Query Terms |
| --- | --- |
| 1 | TITLE-ABS (“tuberculosis” OR “TB) |
| 2 | TITLE-ABS (“C-reactive protein” OR “C reactive protein” OR “CRP”) |
| 3 | TITLE-ABS (“mortality” OR “death” OR “prognosis” OR “survival analysis”) |
| 4 | #1 AND #2 AND #3 |

#### **Table S4. Search Strategy - Cochrane Library**

#### DATE: 10/07/25; Retrieved data: 0

| Query # | Query Terms |
| --- | --- |
| 1 | (“tuberculosis” OR “TB”):ti,ab |
| 2 | “C-reactive protein” OR “C reactive protein” OR “CRP”):ti,ab |
| 3 | (“mortality” OR “death” OR “prognosis” OR “survival analysis”):ti,ab |
| 4 | #1 AND #2 AND #3 |

#### **Table S5. Search Strategy - MEDLINE (via Ovid)**

#### DATE: 10/07/25; Retrieved data: 89

| Query # | Query Terms |
| --- | --- |
| 1 | exp Tuberculosis/ OR tuberculosis.tw. |
| 2 | exp C-Reactive Protein/ OR CRP.tw. |
| 3 | exp Mortality/ OR death.tw. OR prognosis.tw. OR survival.tw. |
| 4 | #1 AND #2 AND #3 |

**Table S6. Search Strategy - Proquest**

#### DATE: 10/07/25; Retrieved data: 92

| Query # | Query Terms |
| --- | --- |
| 1 | (ti(tuberculosis) OR ti(TB) OR ab(tuberculosis) OR ab(TB)) |
| 2 | (ti("C-reactive protein") OR ti("C reactive protein") OR ti(CRP) OR ab("C-reactive protein") OR ab("C reactive protein") OR ab(CRP)) |
| 3 | ti(mortality) OR ti(death) OR ti(prognosis) OR ti("survival analysis") OR ab(mortality) OR ab(death) OR ab(prognosis) OR ab("survival analysis")) |
| 4 | #1 AND #2 AND #3 |

**Table S7. Search Strategy - medRxiv**

#### DATE: 14/07/25; Retrieved data: 297

| Query # | Query Terms |
| --- | --- |
| 1 | (tuberculosis OR TB) AND ("c reactive protein" OR CRP) AND (mortality OR death OR prognosis OR "survival analysis") |
