# Supplementary material for: Prognostic Performance of C-Reactive Protein for Tuberculosis Outcome: Protocol for a Systematic Review and Meta-Analysis
Source: JMIR Res Protoc. 2026 Jun 16;15:e80744. doi: 10.2196/80744 (PMC13271584; doi:10.2196/80744)
Supplement: Checklist 1 [file resprot-v15-e80744-s002.docx]

**Appendix 1 - Reporting checklist for protocol of a systematic review and meta-analysis** **based on the PRISMA-P guidelines**

**Table S1. PRISMA-P (Preferred Reporting Items for Systematic Review and Meta-Analysis Protocols) 2015 checklist; recommended items to address in a systematic review protocol**

| **Section and topic** | **Item No.** | **Checklist item** | **Page** |
| --- | --- | --- | --- |
| **ADMINISTRATIVE INFORMATION** |  |  |  |
| Title: Identification | 1a | Identify the report as a protocol of a systematic review | 1 |
| Title: Update | 1b | If the protocol is for an update of a previous systematic review, identify as such | n/a |
| Registration | 2 | If registered, provide the name of the registry (such as PROSPERO) and registration number | 1 |
| Authors: Contact | 3a | Provide name, institutional affiliation, e-mail address of all protocol authors; provide physical mailing address of corresponding author | 1 |
| Authors: Contributions | 3b | Describe contributions of protocol authors and identify the guarantor of the review | 8-9 |
| Amendments | 4 | If the protocol represents an amendment, state the plan for documenting important protocol amendments | n/a |
| Support: Sources | 5a | Indicate sources of financial or other support for the review | 9 |
| Support: Sponsor | 5b | Provide name for the review funder and/or sponsor | 9 |
| Support: Role | 5c | Describe roles of funder(s), sponsor(s), and/or institution(s) in developing the protocol | 9 |
| **INTRODUCTION** |  |  |  |
| Rationale | 6 | Describe the rationale for the review in the context of what is already known | 2-3 |
| Objectives | 7 | Provide an explicit statement of the question(s) the review will address with reference to participants, interventions, comparators, and outcomes | 3 |
| **METHODS** |  |  |  |
| Eligibility criteria | 8 | Specify the study characteristics (PICO, study design, setting, time frame) and report characteristics (language, publication status) | 3 |
| Information sources | 9 | Describe all intended information sources (electronic databases, contact with authors, trial registers, grey literature) with planned dates of coverage | 3-4 |
| Search strategy | 10 | Present draft of search strategy to be used for at least one database | 4, Appx 2 |
| Study records: Data management | 11a | Describe mechanisms used to manage records and data throughout the review | 4 |
| Study records: Selection process | 11b | State the process for selecting studies through each review phase | 4 |
| Study records: Data collection | 11c | Describe planned method of extracting data from reports | 4 |
| Data items | 12 | List and define all variables for which data will be sought, including assumptions | 4-5 |
| Outcomes and prioritization | 13 | List and define all outcomes for which data will be sought, including prioritization and rationale | 5 |
| Risk of bias in individual studies | 14 | Anticipated methods for assessing risk of bias (at outcome/study level); how this will be used in synthesis | 5-6 |
| Data synthesis | 15a | Describe criteria under which study data will be quantitatively synthesized | 6 |
|  | 15b | If quantitative, describe planned summary measures and methods | 6 |
|  | 15c | Describe proposed additional analyses (e.g., sensitivity, subgroup, meta-regression) | 6 |
|  | 15d | If quantitative synthesis not appropriate, describe summary approach | N/A |
| Meta-bias(es) | 16 | Specify any planned assessment of meta-bias(es) | 6 |
| Confidence in cumulative evidence | 17 | Describe how the strength of the evidence will be assessed (e.g., GRADE) | 6 |
